# Activation of Evolutionarily Young Endogenous Retroviruses Is Implicated in COVID‐19 Immunopathology

**DOI:** 10.1111/gtc.13194

**Published:** 2025-01-19

**Authors:** Reia Yoshida, Hitoshi Ohtani

**Affiliations:** ^1^ Department of Animal Sciences, Graduate School of Bioagricultural Sciences Nagoya University Nagoya Aichi Japan

**Keywords:** COVID‐19, human endogenous retroviruses, innate immunity

## Abstract

The dysfunction of the innate immune system is well‐described as a clinical characteristic of COVID‐19. While several groups have reported human endogenous retroviruses (ERVs) as enhancing factors of immune reactivity, characterization of the COVID‐19‐specific ERVs has not yet been sufficiently conducted. Here, we revealed the transcriptome profile of more than 500 ERV subfamilies and innate immune response genes in eight different cohorts of platelet, peripheral blood mononuclear cells (PBMCs), lung, frontal cortex of brain, ventral midbrain, pooled human umbilical vein endothelial cells (pHUVECs), placenta, and cardiac microvascular endothelial cells (HCMEC) from COVID‐19 patients (total; *n* = 124) and normal samples (total; *n* = 53) using publicly available datasets. While upregulation of ERV subfamilies was found in platelets, PBMCs, and placenta, the immune reactivity was confined to only platelets and PBMCs. It is noteworthy that the evolutionary ages of the upregulated ERV subfamilies detected in platelets and PBMCs were younger than other ERV subfamilies, but the tendency was not seen in the upregulated ERV subfamilies in placenta. The results suggest that only evolutionarily young ERVs can function as enhancing factors of the immune reactivity in COVID‐19 patients. The finding should be instrumental in understanding the COVID‐19 immunopathology.

## Graphical abstract

Dysfunction of the innate immune system is known as a clinical characteristic of COVID‐19. The upregulation of evolutionarily young endogenous retroviruses (ERVs) is implicated in immune reactivity, whereas older ERVs are not.

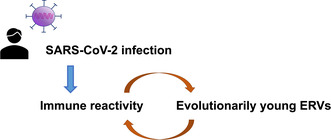

## 1 Introduction

The coronavirus disease 2019 (COVID‐19) has remained a persistent health threat since a global outbreak of severe acute respiratory syndrome coronavirus 2 (SARS‐CoV‐2) (Markov et al. 2023; Minkoff and tenOever 2023). The fact emphasizes that there remains a pressing need to identify the pathogenic factors of COVID‐19. Earlier studies have reported dysfunction of the innate immune system due to aberrant production of interferons and inflammatory cytokines as a clinical characteristic of the disease (Minkoff and tenOever 2023; Villena and Kitazawa 2020; Channappanavar and Perlman 2017; Petrone et al. 2023). This “cytokine storm” is well implicated in COVID‐19 severity and leads to acute respiratory distress syndrome (ARDS) (Mehta et al. 2020; Minkoff and tenOever 2023). Recently, several groups have suggested human endogenous retroviruses (ERVs) as enhancing factors of COVID‐19 severity (Balestrieri et al. 2021, 2023; Temerozo et al. 2022; Petrone et al. 2023; Yin et al. 2021; Kitsou et al. 2021; Grandi et al. 2023). The ERVs, which is one of the transposable elements, make up more than 8% of the host human genome by their repetitive nature (Lander et al. 2001; Cordaux and Batzer 2009; Ohtani and Iwasaki 2021). Since the ERVs are perceived as traces of exogenous viruses, activation of these elements could potentially result in the presence of neoantigens and increased visibility of immune surveillance by the host human cells (Jones et al. 2019). Despite the importance of ERVs in COVID‐19 immunopathology, characterization of specific types of ERVs, which are composed of more than 500 subfamilies, as enhancing factors of the immune reactivity in COVID‐19 has not been sufficiently conducted. We therefore investigated the transcriptome profile of ERV subfamilies and innate immune response genes using RNA‐seq data from various tissues or receptor‐stimulated cells of platelet, peripheral blood mononuclear cells (PBMCs), lung, frontal cortex of brain, ventral midbrain, pooled human umbilical vein endothelial cells (pHUVECs), placenta, and cardiac microvascular endothelial cells (HCMEC).

## 2 Results and Discussion

### 2.1 Expression Patterns of ERV Subfamilies and Innate Immune Genes in Eight Different Cohorts

**FIGURE 1:**
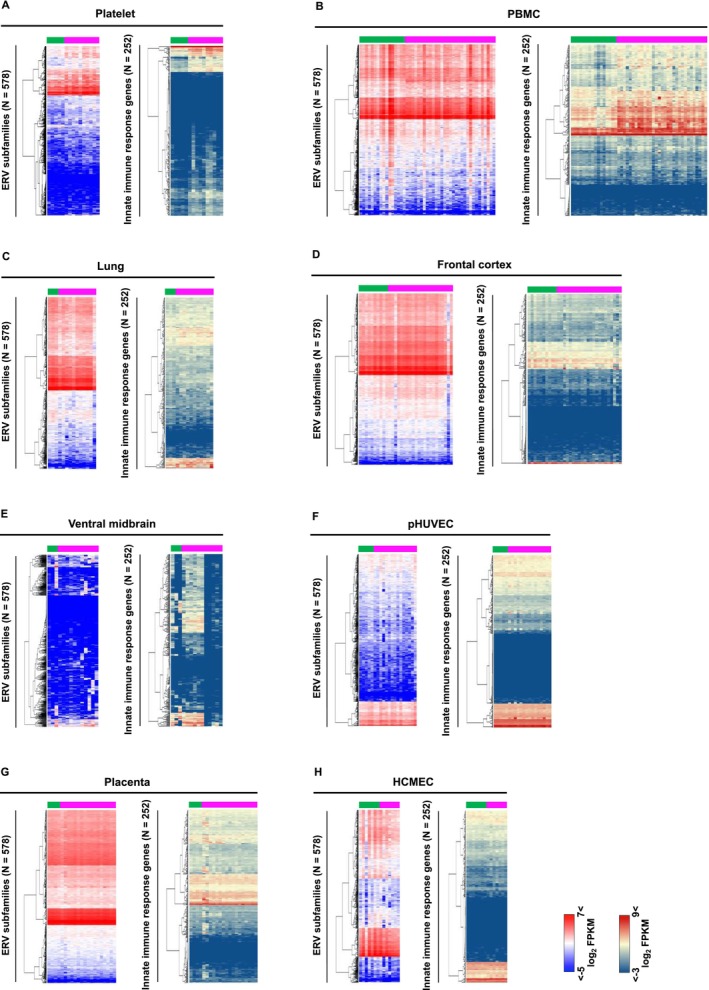
Expression patterns of ERV subfamilies and innate immune genes in eight different cohorts. Heatmaps represent the expression of ERV subfamilies and innate immune genes in (A) platelets, (B) peripheral blood mononuclear cells (PBMCs), (C) lung, (D) frontal cortex of brain, (E) ventral midbrain, (F) pooled human umbilical vein endothelial cells (pHUVECs), (G) placenta, and (H) cardiac microvascular endothelial cells (HCMEC). Green and magenta bars indicate control and COVID‐19 samples, respectively.

To determine transcriptome profile of ERV subfamilies in various types of cells, we first analyzed RNA‐seq data of eight different cohorts from publicly available database (Manne et al. 2020; Togami et al. 2022; Wu et al. 2022; Mavrikaki et al. 2022; Gagliardi et al. 2021; Yang et al. 2024; Gustafson et al. 2022; Barrett et al. 2021) (Figure 1A–H) (Table S1). Since earlier studies defined the aberrant upregulation of ERVs as enhancing factors of dysfunction of host innate immune system (Balestrieri et al. 2021, 2023; Temerozo et al. 2022; Petrone et al. 2023; Yin et al. 2021; Kitsou et al. 2021; Grandi et al. 2023), we next investigated immune reactivity in COVID‐19 patients based on the RNA‐seq data (Figure 1A–H) (Table S2). Although cell type‐specific expression patterns were observed among the eight cohorts, it is not suitable to conduct comparative analyses on these samples, which are produced by different conditions. Total RNA samples of platelets, PBMCs, lung, frontal cortex of brain, and placenta were extracted from each tissue of COVID‐19 patients. On the other hand, pHUVECs and HCMEC samples were obtained from ex vivo experiments by exposure them to COVID‐19 patient plasma and platelet‐released factors from COVID‐19 patients, respectively (Gustafson et al. 2022; Barrett et al. 2021). The ventral midbrain samples were prepared following SARS‐CoV‐2 infections of midbrain dopamine neurons derived from brain donors (Yang et al. 2024). We therefore decided to focus on the association of expression changes between ERVs and innate immune response genes in each independent study, as we previously reported (Ohtani et al. 2020).

### 2.2 The Association of ERVs and Immune Reactivity Was Observed in Platelet and PBMCs, but Not Placenta

**FIGURE 2:**
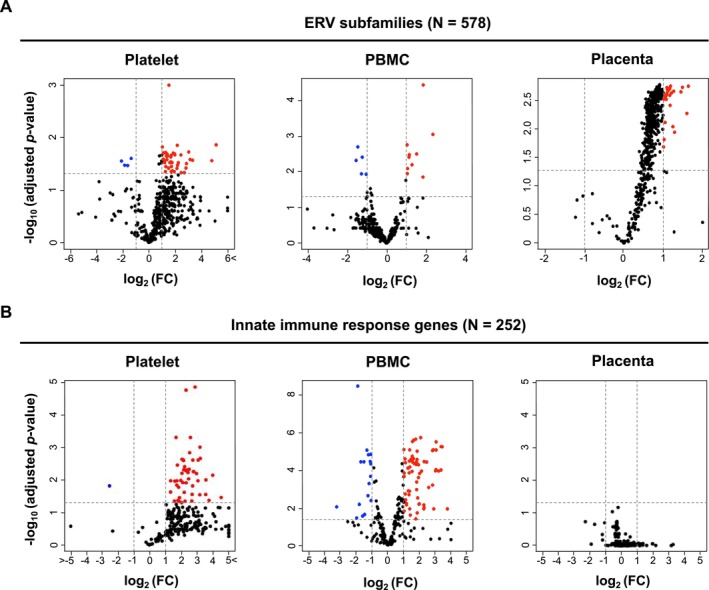
Expression changes of ERV subfamilies and innate immune genes in the three tissues. The volcano plots represent expression changes of (A) ERV subfamilies and (B) innate immune response genes in platelets, PBMCs, and placenta. The log_2_ FC values and −log_10_ (*p* value) were calculated by comparison with expression of genes in control with COVID‐19 patients. Black dashed lines are the threshold of the Welch *t* test with Holm's method *P* value < 0.05 (horizontal) or FC > 2 (vertical). The red and blue points show upregulated and downregulated ERV subfamilies in COVID‐19 patients, respectively.

Although cell type‐specific basal expression patterns were constructed (Figure 1A–H), the statically significant upregulation of ERV subfamilies in COVID‐19 patients by comparison with normal samples was detected only in three kinds of tissues, platelet, PBMCs, and placenta (Figure 2A). Consistent with earlier research (Guo, Zhao, and You 2024), certain ERV subfamilies, such as LTR7Y and MER52‐int, were also identified in our study using PBMCs (Table S1). Although the underlying mechanisms responsible for the upregulation were unclear, the ERVs may be regulated by promoter regions that are bound by transcription factors upon activation of innate immune systems by the infection of viruses as described in previous reports (Ohtani et al. 2024; Chuong, Elde, and Feschotte 2016). Earlier studies defined the aberrant upregulation of ERVs as enhancing factors of dysfunction of the host innate immune system (Balestrieri et al. 2021, 2023; Temerozo et al. 2022; Petrone et al. 2023; Yin et al. 2021; Kitsou et al. 2021; Grandi et al. 2023), we next examined immune reactivity in the three tissues based on the RNA‐seq data. As consistent with previous studies, the robust upregulations of innate immune response genes were confirmed in COVID‐19 patients from platelet and PBMCs (Figure 2B) (Manne et al. 2020; Togami et al. 2022). On the other hand, contrary to the expectation, we could not find upregulation in the case of placenta in spite of the fact that a large number of ERV subfamilies were upregulated in the tissue (Figure 2A,B).

### 2.3 Upregulated Evolutionarily Young ERV Subfamilies May Be Involved in the Immune Reactivity in COVID‐19

**FIGURE 3:**
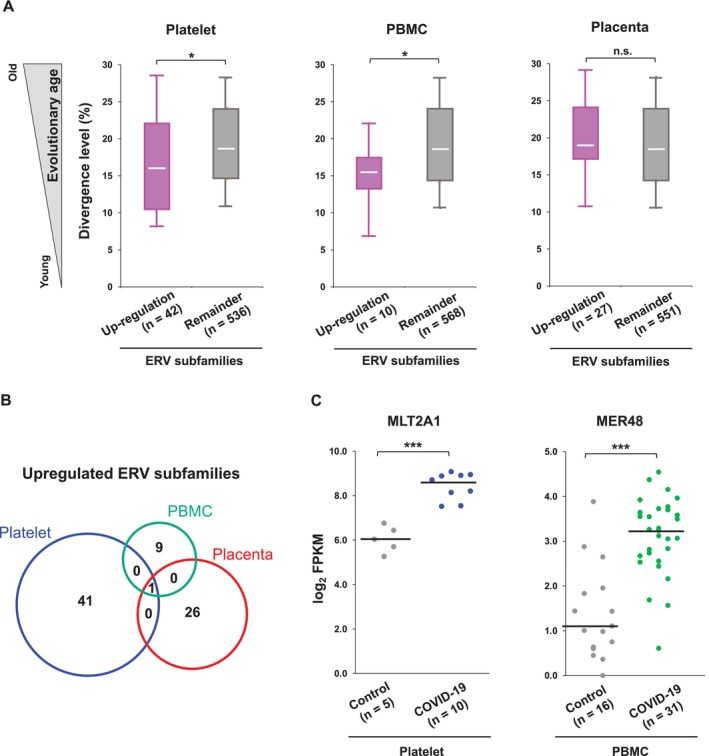
Evolutionarily young ERV subfamilies were upregulated in platelet and PBMC, but not placenta. (A) The distribution of divergence levels in ERV subfamilies. Each box represents the data between the 25th and 75th quartiles. The whiskers are drawn down to the 10th percentile and up to the 90th percentile. *P* values were obtained using a two‐tailed Mann–Whitney U test between the two groups. *p < 0.05. (B) ERVs overlapping among upregulated ERV subfamilies in platelet, PBMC, and placenta. The group of upregulation ERV subfamilies refers to in Figure 2A in each tissue. (C) Expression levels of representative platelet‐specific upregulated ERV subfamily MLT2A1 and PBMC‐specific upregulated ERV subfamily MER48. Dots represent each sample. Black lines represent median values. *P* values were obtained using the Welch *t* test with Holm's method between the two groups. ***p < 0.001.

As we and other groups earlier reported that evolutionarily young ERVs might be responsible for the reactivity of innate immune response in cancer cells (Guler et al. 2017; Ohtani et al. 2018, 2020), we decided to investigate the evolutionary ages of the COVID‐19‐specific upregulated ERV subfamilies identified in platelet, PBMC and placenta (Figure 3A). The evolutionary ages of ERV subfamilies were estimated using genetic divergence level deposited in RepeatMasker database, which is considered indices of evolutionary age of repetitive elements (Moorjani et al. 2016). While the COVID‐19‐specific upregulated ERV subfamilies detected in platelet and PBMCs showed significantly lower divergence levels than the remainder of ERV subfamilies, there were no statistical difference between the upregulated ERV subfamilies detected in placenta and remainders (Figure 3A). This result is in line with our previous studies in vitro (Ohtani et al. 2018, 2020), which reported evolutionarily young ERV‐derived RNAs as potential triggers for the activation of the innate immune system, but not evolutionarily old ERV‐derived RNAs. Since the upregulated ERV subfamilies were not shared between platelet and PBMC except for LTR7Y, which is also identified in placenta (Figure 3B), distinct kinds of evolutionarily young ERV subfamilies may function redundantly as neoantigen for the immune reactivity, such as MLT2A1 and MER48 (Figure 3C).

Importantly, several previous studies have also reported aberrant expression of HERV‐K and HERV‐W, known as evolutionarily young ERV subfamilies, in PBMCs and bronchoalveolar lavage fluid samples from COVID‐19 patients (Bao et al. 2024; Kitsou et al. 2021; Evans, Saraph, and Tokuyama 2024). Although the potential immune activation competence of the evolutionarily young ERVs has not yet been demonstrated, the aberrant expression of evolutionarily young ERVs may be thought of as being an infection by exogenous viruses termed “viral mimicry” (Jones et al. 2019). However, further studies are necessary to understand the evolutionarily young ERVs‐mediated pathogenesis of COVID‐19.

### 2.4 Expression Patterns of Two ERV Subfamilies Were Associated With Disease Severity of COVID‐19

**FIGURE 4:**
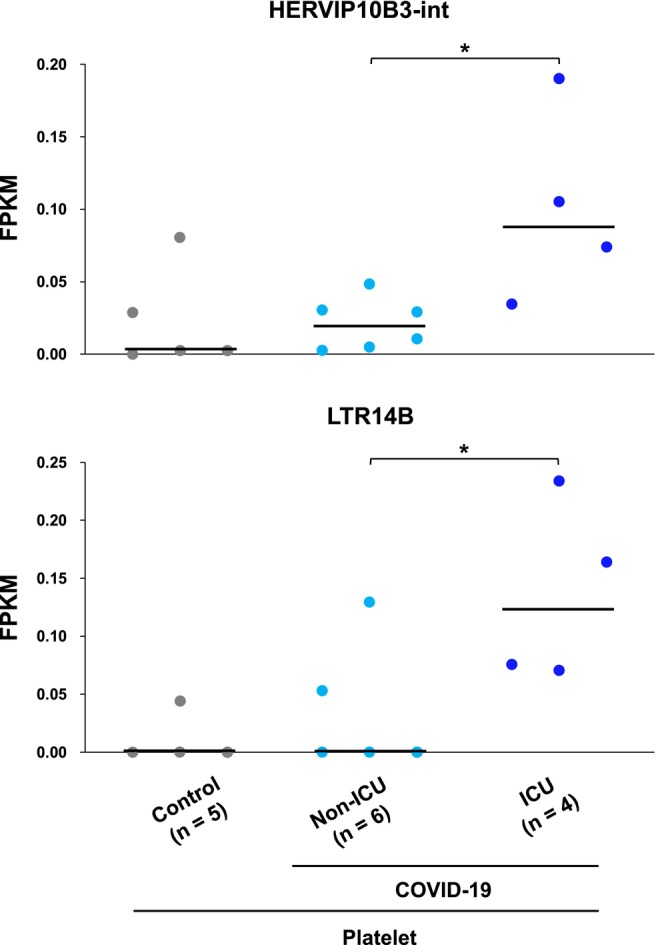
HERVIP10B3‐int and LTR14B were upregulated in severe COVID‐19 cases. Expression levels of HERVIP10B3‐int and LTR14B ERV subfamilies in healthy controls and each disease stage of COVID‐19. Dots represent each sample. Black lines represent median values. *P* values were obtained using the Welch *t* test between the two groups. *p < 0.05.

Since COVID‐19 severity data was available in the platelet cohort, we investigated ERV subfamilies with differential expression patterns between non‐ICU (*n* = 6) and ICU (*n* = 4) COVID‐19 patients (Figure 4). The HERVIP10B3‐int and LTR14B subfamilies were upregulated in severe COVID‐19 compared to other cases. Although the ERVs may be of use as novel biomarkers to predict disease progression, further large‐scale studies are necessary to identify potential biomarkers for evaluating the severity of COVID‐19.

## 3 Conclusion

This work revealed expression profiles of ERV subfamilies in eight different cohorts and characterized them based on evolutionary ages. Although activation of evolutionarily young ERVs may be involved in the mechanism of well‐described COVID‐19‐specific immune reactivity, evolutionarily old ERVs may not have the capability. The findings should be valuable to understand the COVID‐19 immunopathology.

## 4 Methods

### 4.1 Transcriptome Data of COVID‐19

RNA‐seq data of COVID‐19 patients and control samples were downloaded from a publicly available database (Manne et al. 2020; Togami et al. 2022; Wu et al. 2022; Mavrikaki et al. 2022; Gagliardi et al. 2021; Yang et al. 2024; Gustafson et al. 2022; Barrett et al. 2021). The data from platelet (COVID‐19; *n* = 10, control; *n* = 5), PBMCs (COVID‐19; *n* = 31, control; *n* = 16), lung (COVID‐19; *n* = 11, control; *n* = 3), frontal cortex of brain (COVID‐19; *n* = 22, control; *n* = 10), ventral midbrain (COVID‐19; *n* = 11, control; *n* = 3), pooled human umbilical vein endothelial cells (pHUVECs) (COVID‐19; *n* = 15, control; *n* = 5), placenta (COVID‐19; *n* = 17, control; *n* = 4), and cardiac microvascular endothelial cells (HCMEC) (COVID‐19; *n* = 7, control; *n* = 7) were obtained from NCBI Gene Expression Omnibus (GEO; http://www.ncbi.nlm.nih.gov/geo/) under the accession numbers (Table S3). The information of clinical status of these samples is also deposited under the accession numbers.

### 4.2 Expression Analysis of ERVs and Coding Genes

Sequencing reads obtained from the RNA‐seq were aligned to the human GRCh38/hg38 reference genome using HISAT2 (Kim et al. 2019). The innate immune response genes were defined based on positive regulation of innate immune response (GO:0045089) in Gene Ontology (Gene Ontology Consortium et al. 2023). The genomic positions of ERV elements were acquired from RepeatMasker database (http://www.repeatmasker.org). The ERVs located in intergenic regions were used for this analysis to determine the transcripts emanating from the ERVs, as described in our previous studies (Ohtani et al. 2018). The expression of 578 ERV subfamilies was calculated using the sum of aligned read counts of ERV copies on each subfamily. FPKM (fragments per kilobase per million mapped reads) values were calculated using uniquely mapped reads outputted from featureCounts. To avoid differences in mapping efficiency on the repetitive elements affecting the results, we used fold‐changes relative to control samples. Source codes are below: hisat2 ‐x /grch38/genome −1 Sample_1.fastq −2 Sample_2.fastq ‐p 14 | samtools view ‐bS ‐ | samtools sort ‐T Sample.tmp ‐O bam ‐o Sample.bam. featureCounts ‐p ‐s 2 ‐T 5 ‐t exon ‐g gene_id ‐a Repeatmasker.gtf ‐o Sample.tsv Sample.bam ‐‐primary ‐Q 20 ‐‐ignoreDup.

### 4.3 Acquisition of Evolutionary Ages of ERV Subfamilies

Evolutionary ages of ERV subfamilies were estimated based on the average genetic divergence levels of each copy, using “div.” values in the annotation file downloaded from the RepeatMasker database (http://www.repeatmasker.org), which is a gold standard index to estimate the evolutionary ages.

## Author Contributions

**Reia Yoshida:** data curation, formal analysis, investigation. **Hitoshi Ohtani:** conceptualization, data curation, formal analysis, investigation, funding acquisition, visualization, project administration, supervision, writing – original draft, writing – review and editing, validation.

## Conflicts of Interest

The authors declare no conflicts of interest.

## Supporting information


**Table S1.** FPKM values of ERV subfamilies.
**Table S2.** FPKM values of innate immune response genes.
**Table S3.** Transcriptome dada of COVID‐19 and control.

## Data Availability

The data that supports the findings of this study are available in the Supporting Information of this article.
